# Effect of Combination of Time and Temperature on Quality Characteristics of Sous Vide Chicken Breast

**DOI:** 10.3390/foods11040521

**Published:** 2022-02-11

**Authors:** Endrit Hasani, Barbara Csehi, Lívia Darnay, Márta Ladányi, István Dalmadi, György Kenesei

**Affiliations:** 1 Department of Livestock Products and Food Preservation Technology, Institute of Food Science and Technology, Hungarian University of Agriculture and Life Sciences, Ménesi út 43-45, 1118 Budapest, Hungary; csehi.barbara@uni-mate.hu (B.C.); dalmadi.istvan@uni-mate.hu (I.D.); kenesei.gyorgy@uni-mate.hu (G.K.); 2 Department of Food Technology and Biotechnology, Faculty of Agriculture and Veterinary, University of Prishtina, 10000 Prishtina, Kosovo; 3 Department of Food Hygiene, University of Veterinary Medicine Budapest, István u.2., 1078 Budapest, Hungary; darnay.livia@univet.hu; 4 Department of Applied Statistics, Institute of Mathematics and Basic Science, Hungarian University of Agriculture and Life Sciences, Villányi út 29-43, 1118 Budapest, Hungary; ladanyi.marta@uni-mate.hu

**Keywords:** sous vide, poultry meat, cooking loss, color, shear force, protein solubility, lipid oxidation, pasteurization values

## Abstract

The use of minimal thermal processing techniques such as sous vide technology to improve the quality of meat-based foods has gained a special focus in recent years. A proper combination of temperature and time parameters in sous vide processing plays an important role in the water-holding capacity, texture properties, and juiciness of the meat. The present study aimed to assess the impact of the one-step and two-step sous vide processing on different quality properties of chicken breast with special emphasis on the cooking loss, color, texture properties, protein solubility, and lipid oxidation. According to the results, chicken breast treated with a two-step temperature (50 and 60 °C) showed improved texture parameters (shear force, hardness, chewiness, and gumminess), lower cooking loss, acceptable redness values, and decreased lipid oxidation levels than the chicken breast treated with the one-step temperature of 60 °C. Moreover, the two-step sous vide technique revealed significantly higher total protein solubility of the chicken breast than the one-step sous vide. Based on pasteurization values, the two-step sous vide technique was equally safe as the one-step sous vide technique for vegetative cells’ inactivation for the main pathogens of interest (*C. perfringens* and *L. monocytogenes*).

## 1. Introduction

The global consumption of poultry meat is gradually increasing because of its beneficial effects on human health and the low price compared to the rest of the meats. The high level of protein, complete amino acid composition, essential micronutrients, and low levels of fat in poultry meat provide a healthy diet for different groups of people [1]. These features reduce the risk of obesity and prevent malnutrition and cardiovascular diseases in humans [2]. However, poultry meat consumption may be limited among the elderly because of the mastication and swallowing challenges due to different oral impairments [3,4]. Thus, in the case of chicken meat, breast muscles tend to show higher toughness and crumbliness as compared to chicken legs’ muscles [5,6]. In this context, the food industry needs to develop novel poultry-based ready-to-eat products with an improved texture and high nutritional value to meet these demands.

Sous vide cooking is a minimal processing technology that has gained popularity in the food industry, catering sector, and restaurants in recent years [7,8]. Sous vide in French means “cooking under vacuum” and refers to mild heat treatment of food in a water bath under strictly monitored parameters (temperature and time) using thermostable vacuum pouches [9]. This technique provides better control over the texture of the product, inhibits lipid oxidation, improves sensorial characteristics, and minimizes protein denaturation depending on treatment conditions [10].

Identification of optimal temperature and time duration is often challenging in sous vide processing to achieve desired attributes of cooked meat such as tenderness or juiciness. Most researchers have studied only the influence of different combinations of a single temperature and time in sous vide processing on the quality attributes of beef [11,12,13], pork [14], turkey [15], and chicken [16,17]. On the other hand, several authors reported using additional pretreatments such as freezing technology [12] or thawing [18] before sous vide to achieve meat tenderization. Botinestean et al. [12] found that short-term freezing of beef steaks at −20 °C resulted in comparable shear force, hardness, and chewiness values with sous vide cooking alone. Ji et al. [18] concluded that freeze-thawing and freezing applied prior to sous vide significantly decreased the shear force of pork meat. However, from an economic perspective, these attempts do not seem feasible because they impose extra processing costs. Furthermore, including pretreatments such as thawing in sous vide processing may increase the weight loss of cooked meat [18]. Therefore, plant-derived exogenous proteases such as papain, actinidin, zingibain, and bromelain were also tested [19,20,21,22]. These alternatives have significant proteolytic activity and proved effective to increase the tenderness of the treated meat [23,24,25]. However, if fresh plant extract is applied in meat, it could produce non-desirable off-flavors [26,27].

On the other hand, meat has its native endogenous proteolytic enzymes, the highest activity of which has been reported to be at temperatures between 40–50 °C [28]. Previous studies reported that the high activity of proteolytic enzymes at these temperatures is responsible for higher desmin degradation, which indicates an extension of meat tenderization [14,29]. The proteolytic activity of the calpain-1, calpain-2, and cathepsin B enzymes responsible for desmin degradation is more intensive between 40 and 55 °C and negligible at 60 °C and above [29]. Therefore, the inclusion of a low temperature as a first-step temperature in the sous vide technique may improve meat tenderness and other quality characteristics. There are no studies in the literature that examine the effect of sous vide with a two-step temperature on chicken breast muscles. Thus, this technique may provide an alternative cooking method to develop novel poultry-based ready-to-eat foods with an improved texture. Furthermore, it may provide valuable data to the food industry to use as a reference. Therefore, the objective of the present study was to investigate the effect of sous vide using the one-step temperature of 60 °C and the two-step temperatures (50 °C + 60 °C) applied in different time ratios of the same total treatment times, on physicochemical characteristics, texture attributes, lipid oxidation, and protein solubility of chicken breast muscles (*pectoralis major*). In addition, the pasteurization values of the chosen sous vide treatments were also calculated.

## 2. Materials and Methods

### 2.1. Experimental Design

*Pectoralis major* muscles (24 h post-mortem, pH_24_ = 5.83 ± 0.12) were obtained from a slaughterhouse in Budapest, Hungary, and were kept overnight at a refrigerated temperature of 4 °C until sample preparation. The chicken breasts were trimmed of external fat and connective tissues and cut into uniform pieces of 2 ± 0.2 cm thick, weighing about 130 ± 2.4 g each. The samples were vacuum packaged in 90-μm PA/PE pouches (200 mm × 250 mm) with a vacuum machine (Multivac C100, MULTIVAC Sepp Haggenmüller SE & Co. KG, Wolfertschwenden, Germany) and were randomly assigned in one of the six groups of different temperature and time processing combinations (Table 1).

In the one-step sous vide treatments (T1 and T4), chicken breasts were cooked at 60 °C for 120 and 180 min, while in the two-step sous vide, temperatures of 50 °C and 60 °C were combined at different time ratios of the same total treatment times (Table 1). Three replicates were conducted for each processing treatment using a two-way (completely randomized) design. The two factors were total treatment time (two levels: 120 and 180 min) and treatment time ratio (three levels: 0:1, 1:2, 1:1).

Sous vide processing was performed in two thermostatic water baths (Labor Müszeripari Müvek LP507/01). A T-type thermocouple inserted at the geometric center of the muscle was used to monitor the core temperature of the samples during thermal processing. The data logger recorded the temperature readings during processing at intervals of 2 s (scanning time) with a ±0.1 °C accuracy. After that, the cooked chicken breasts were put in ice-cold water at 1 °C for 60 min to cool down and then were maintained overnight at refrigerated conditions (2 °C) to reach a temperature of less than 4 °C for a period of 6 h, according to the recommended guidelines [30]. Physicochemical properties (moisture content, cooking loss, color attributes, texture properties), lipid oxidation, protein solubility, and pasteurization values were determined on the following days after thermal processing.

### 2.2. Pasteurization Values

The calculation of pasteurization values of each group was performed by integration of the time–temperature profile curves derived from the recorded time–temperature readings during treatments, according to the equation below:

(1) $$P_{T_{ref}}^{z}=\int_{0}^{t}10^{\left(T-T_{ref}\right)/z}dt$$

In Equation (1), *t* indicates the heating time (min), *T* refers to the measured core temperature (°C) of the muscle during treatment, *T_ref_* indicates the reference temperature, and *z* value refers to the number of degrees of temperature required to increase the thermal death rate of the microorganism by a factor of 10. Three reference temperatures *T_ref_* (60, 70, and 80 °C) for three target pathogens, with specific z-values of 6.74 °C (*C. perfringens*) [31], 10 °C (*L. monocytogenes*) [32], and 13 °C (*C. botulinum* spores) [33], respectively, were used to calculate the pasteurization values of the treatments included in the study. Calculations were done based on five independent batches for each group treatment.

### 2.3. Physicochemical Analysis

#### 2.3.1. Moisture Content and Cooking Loss

The moisture content of chicken breasts was measured in triplicate using the standard AOAC International 950.46 method [34]. Briefly, around 4 g of cooked chicken breast were weighed and subjected to drying in an air-forced oven (Labor Müszeripari Müvek, Budapest, Hungary) at 105 °C for 16 h. Cooking loss was calculated from the weight difference between the raw and cooked chicken breasts.

#### 2.3.2. Color Measurement

The color attributes of chicken breasts were determined using the CIELAB [35] scoring system. A CR-410-type colorimeter (Konika Minolta Sensing Inc., Osaka, Japan) was used to measure the lightness, redness, and yellowness values of the samples after white calibration of the instrument. Five parallel readings were performed for each sample (*n* = 3). The method described by Knispel [36] was used to calculate the total color difference (ΔE) between groups using the obtained values of lightness L*, redness a*, and yellowness b*, based on the equation below:

(2) $$\Delta E=\;\sqrt{{\left(L^{*}-L_{0}^{*}\right)}^{2}+{\left(a^{*}-a_{0}^{*}\right)}^{2}+{\left(b^{*}-b_{0}^{*}\right)}^{2}}$$

#### 2.3.3. Texture Properties

The texture of cooked samples was analyzed through texture profile analysis (TPA) following the method applied by Bourne [37]. Twelve chicken breast cores per treatment in duplicates (diameter = 12 mm, height = 12 mm), previously tempered at 4 °C, were compressed to 70% of their original height in two cycles with a P/35 cylinder probe (35 mm diameter) using a TA.XT Plus texture analyzer (Stable Micro System, Surrey, United Kingdom). A load cell (500 N) was applied at a speed of 2 mm s^−1^ from which maximum force-time deformation curves were obtained. Texture Expert software for Windows (Stable Micro System) was used for processing data to assess the TPA attributes: hardness, springiness, gumminess, cohesiveness, and chewiness. For shear force analysis, samples were cut on a slab shape in the size of 15 mm × 15 mm × 50 mm (width, thickness, length) and cut perpendicular to the orientation of fibers with a Warner–Bratzler knife blade with a flat end at 2 mm/s speed. The obtained peak force (N) was registered as a shear force value. Six parallel measurements were performed for each sample (*n* = 3).

### 2.4. Lipid Oxidation

To determine the lipid oxidation of chicken breasts, the thiobarbituric Acid Reactive Substances (TBARS) values were analyzed using the method from Dias et al. [38] with few modifications. The sample (5 g) was homogenized (Digital Ultra-Turrax, Staufen, Germany) for 2 min with 20 mL of 5% trichloroacetic acid (TCA) and 0.5 mL of the artificial antioxidant 0.15% butylated hydroxytoluene (BHT, Sigma-Aldrich, Munich, Germany). After that, centrifugation of the homogenized samples was performed at 5000× *g* for 10 min. The obtained supernatants were filtered using Whatman No. 1 filter paper into 25-mL volumetric flasks and adjusted to 25 mL with 5% TCA. Two milliliters of the filtrate were mixed with two milliliters of 0.08% thiobarbituric acid (TBA) in glass tubes and were heated in a water bath at 95 °C for half an hour. Solutions in glass tubes were cooled down to room temperature, vortexed, and then absorbances were measured against a blank (mixing 2 mL of 5% TCA and 2 mL of 0.08% TBA) at 532 nm by U 2900 UV-visible spectrophotometer (Hitachi Ltd., Tokyo, Japan). TBARS values represented the mean of triplicate measurements for each sample (*n* = 3) and were reported as mg malondialdehyde/kg of meat sample [39,40].

### 2.5. Protein Solubility

Protein solubility parameters were assessed using the method applied by Warner et al. [41]. One gram of sample was homogenized in triplicates with 10 mL of ice-cold 25 mM potassium phosphate buffer (pH = 7.2) by a Digital Ultra-Turrax homogenizer (Staufen, Germany) for the extraction of sarcoplasmic proteins. Homogenates were left for 20 h in refrigerated conditions (4 °C) and then were centrifuged (1500× *g* for 20 min) at 4 °C. Protein concentrations of the obtained supernatants were assessed following the Bradford method [42] using the BSE (bovine serum albumin) as a standard. Similarly, total protein solubility was determined by homogenizing 1 g of sample with 10 mL of 0.55 M potassium iodide and 0.05 M potassium phosphate buffer (pH = 7.2). Myofibrillar protein solubility was calculated as the difference between total protein solubility and sarcoplasmic protein solubility.

### 2.6. Statistical Analysis

The experimental data analysis was performed using IBM SPSS (Version 27.0, Armonk, NY, USA, 2020). Two-way multivariate MANOVA models were performed for moisture content and cooking loss, for color parameters (L*, a*, b*, and ΔE), for protein solubility parameters (sarcoplasmic, myofibrillar, and total protein solubility), and for TPA parameters (hardness, cohesiveness, springiness, gumminess, chewiness), and two-way ANOVA models for TBARS and shear force. The two factors analyzed were total treatment time (two levels: 120 and 180 min) and the time ratio (three levels: 0:1, 1:2, and 1:1). Normality of the residuals was tested by the Kolmogorov–Smirnov test (*p* > 0.05). Homogeneity of error variances was accepted by Levene’s test (*p* > 0.05) except in the case of gumminess (*p* < 0.05). Multivariate overall test was evaluated based on the unexplained variance ratio (Wilk’s lambda) and in the case of significant overall result, follow-up univariate ANOVA was performed using Bonferroni’s correction to avoid familywise Type I error inflation. At the end, Tukey’s post hoc tests were run if homogeneity of variances was satisfied and Games–Howell’s method was used when this assumption was violated.

## 3. Results

### 3.1. Investigation of Pasteurization Values of the Sous Vide Treatments

The pasteurization process has an important role in the sensorial attributes, nutritional value, and microbial stability of sous vide meat products [15,16]. Pasteurization values (*p*-values) are defined as the duration of the pasteurization process to achieve a particular reduction of a target pathogen at a specified temperature. To calculate pasteurization values of sous vide treatments, three target pathogens associated with sous vide meat products were considered: *C. perfringens* ($P_{60}^{6.74}$), *L. monocytogenes* $\left(P_{70}^{10}\right),$ and *C. botulinum* spores ($P_{80}^{13}$). Pasteurization values ($P_{60}^{6.74}$, $P_{70}^{10}$, $P_{80}^{13}$) of the studied sous vide treatments derived from the recorded time–temperature profiles are presented in Table 2. As expected, chicken breasts treated with the one-step temperature obtained higher pasteurization values than the chicken breasts treated with the two-step temperature within the same total treatment time.

For pasteurization of sous vide foods, the European Chilled Food Federation (ECFF) [33] recommends at least a heat treatment equivalent to 70 °C for 2 min to ensure a 6-log reduction of *L. monocytogenes* (z-value = 10 °C). According to the results presented in Table 2, all the studied sous vide treatments achieved sufficient inactivation of *L. monocytogenes* from chicken breast. Previous studies have also reported successful inactivation of *L. monocytogenes* and other vegetative microorganisms by sous vide treatments [43,44]. On the other hand, both one-step and two-step sous vide treatments were insufficient to pasteurize chicken breast for *C. botulinum* spores as the pasteurization values ($P_{80}^{13})$ were lower than 26 min [34]. This poses a potential safety risk; thus, it requires proper storage at temperatures lower than 3 °C or at freezing temperatures to prevent the spore multiplication of *C. botulinum* [45].

*Clostridium perfringens* is a mesophilic spore-forming bacteria usually used as an indicator of thermal efficiency in sous vide treatments. From the thermal inactivation data of *C. perfringens* (D_60_ = 5.3 min and z-value = 6.74 °C) [32], food products need to be pasteurized with a heating process equivalent to 60 °C for 32 min to achieve a 6-log vegetative cell reduction. From our results, it can be seen that all the studied sous vide treatments achieved the minimum pasteurization value of $P_{60}^{6.74}$ = 32 min.

### 3.2. Physicochemical Analysis

#### 3.2.1. Moisture Content and Cooking Loss

A comparison of the physicochemical properties of chicken breast (moisture content, cooking loss, and color attributes) between the studied sous vide treatments are displayed in Table 3. In the cases of moisture content and cooking loss, the two-way MANOVA overall result was significant for both factors (total treatment time: Wilk’s lambda = 0.07, *p* < 0.001; time ratio: Wilk’s lambda = 014, *p* < 0.001) with insignificant interaction (*p* = 0.22). The follow-up univariate ANOVA using Bonferroni’s correction revealed significant results for both variables and both factors (all with *p* < 0.001).

Higher moisture content was observed when the application time of the low temperature (50 °C) was increased from 0 to 90 min in the 180-min sous vide treatments, with a significant increase only between T4 and T6 (*p* < 0.05). On the other hand, no significant increase in moisture content was observed between treatment time ratios within the 120-min sous vide treatments (T1, T2, T3) (*p* > 0.05). Our results are in agreement with those reported by other authors [15,46]. Bıyıklı et al. [15] found that moisture content was significantly higher in turkey cutlets cooked at lower sous vide cooking temperatures. The moisture content of meat depends on the degree of thermal protein denaturation, which causes shrinkage of muscle fibers [47], leading to a volume reduction and, consequently, water loss at high cooking temperatures.

Cooking losses during heat treatment contribute to a considerable share of food wastes in households and catering sector [48]. Sous vide presents a valuable method to reduce food waste by the combination of optimal temperature and time duration of cooking in heat-stable pouches. In our study, the cooking loss of chicken breasts cooked with different sous vide treatments ranged from 11.61% to 16.35% (Table 3). Chicken breasts cooked with the two-step sous vide treatment T3 had significantly lower cooking loss than the chicken breasts cooked with the one-step sous vide treatment T1 (*p* < 0.05). Low cooking loss correlates with higher juiciness, which has a positive impact on consumer preference for chicken meat [49]. A similar result was observed also between the 180-min one-step (T4) and two-step sous vide treatments (T5 and T6). Apparently, the application of the lower temperature (50 °C) in the two-step sous vide had a positive effect on lowering cooking loss and thus improving the juiciness of the chicken breast. In agreement with this result, Hwang et al. [46] reported that cooking loss of sous vide pork loins was lower when cooked at 50 °C compared to 55 and 60 °C. Zielbauer et al. [50] reported lower values of cooking loss compared with the trend during a long time of cooking pork at the temperature of 51 °C. This could be explained by collagen solubilization under denaturation, which results in gel formation and higher water content in meat [51].

#### 3.2.2. Color Measurement

In Table 3 are presented the instrumental color properties (L*, a*, b*, and ΔE) of the chicken breast cooked in different combinations of temperature and time. Two-way MANOVA overall result was significant for both factors (total treatment time: Wilk’s lambda = 0.23, *p* < 0.01; time ratio: Wilk’s lambda = 0.003, *p* < 0.001) with significant interaction (*p* < 0.001). The follow-up univariate ANOVA using Bonferroni’s correction revealed significant results for lightness (*p* < 0.05) and ΔE (*p* < 0.05) in the case of total treatment time and an insignificant result for a* (*p* = 0.91) and b* (*p* = 0.51). Meanwhile, univariate ANOVA resulted in a significant time ratio effect for all the four parameters (*p* < 0.001). The interaction was significant only for b* (*p* < 0.05). 

Color is one of the main indicators in consumer perception of meat quality and safety [52]. Changes in the color properties of meat products treated with heat can be attributed to myoglobin denaturation and oxidation, Maillard reactions, packaging, or pH [51]. Compared to conventional cooking, sous vide-cooked meat obtains higher redness and lightness, which is due to myoglobin changes during denaturation, with deoxymyoglobin being the most resistant to protein denaturation from the three forms of myoglobin [53]. The present study showed that the two-step sous vide-treated samples had significantly lower lightness (L* value) than the one-step sous vide-treated samples within the same total treatment time (*p* < 0.05). This can be explained by the fact that higher temperatures can increase the protein denaturation process, which results in a higher brightness of meat [14]. Cooked chicken breast can be classified into three categories based on the degree of lightness: pale (L* < 53), normal (L* = 46–53), and dark (L* < 46) [54]. Based on the obtained lightness values, all the studied sous vide treatments belonged to the pale appearance classification group (L* > 53).

Redness values were between 1.34 and 2.46. The present study found no significant difference in redness when the total treatment time was increased from 120 min (T1) to 180 min (T4) at 60 °C (*p* > 0.05). In agreement with this result, Haghighi et al. [55] reported that redness values of sous vide chicken breasts were not affected when the cooking time was increased from 60 min to 150 min at the temperature of 60 °C. In contrast, Park et al. [56] reported that chicken breasts cooked at 60 °C for 180 min had lower redness compared to those cooked for 120 min. Our study revealed that chicken breast cooked with the 180-min sous vide had significantly higher redness values with a longer application time of the low temperature of 50 °C in the sous vide treatment. This might be attributed to myoglobin pigment concentration, which is presumed to be higher in the two-step sous vide-treated samples because its denaturation starts between 55 and 65 °C [53]. The pink color in poultry meat has been shown to be the first indicator that consumers check for a properly cooked product [57]. The degree of the pink color on chicken breast can be evaluated using a subjective pink threshold of a* = 3.8 [58]. In the present study, all the sous vide-treated samples obtained redness values under this threshold level.

The yellowness values of the chicken breasts cooked with the two-step temperature were significantly higher than those processed with the one-step temperature within the same total treatment time (*p* < 0.05). This might be due to a lower exposure of meat to the temperature of 60 °C during cooking in the two-step sous vide treatments (40–90 min) in comparison with the one-step sous vide treatments (120 and 180 min) (Table 3). 

Results of the total color difference (ΔE) between raw and cooked chicken breasts are presented in Table 3. The highest total color difference (ΔE) was obtained for treatment T4 (30.06) and the lowest, for treatment T2 (28.64). According to Mokrzycki and Tatol [59], an observer notices two different colors when ΔE > 5. In this context, the authors believe that for a better understanding of changes in the color attributes of the samples it is more common to evaluate the total color difference (ΔE) between the studied sous vide treatments. Total color difference (ΔE) results between sous vide treatments were classified based on five categories as described by Mokrzycki and Tatol [59] and are presented below in Table 4.

According to the study of Tomasevic et al. [60], a clear color difference between meat products is noticeable if ΔE > 3.5. From the pairwise comparison between the 120-min sous vide treatments, a clear color difference can be noticed between the chicken breast cooked with the one-step temperature treatment (T1) and the two-step temperature treatment (T3) where the total color difference (ΔE) was 3.94 (Table 4). Similar results were obtained for total color difference (ΔE) between the 180-min one-step (T4) and two-step sous vide-treated chicken breasts (T5, T6) (ΔE > 3.5). On the other hand, the total color difference between chicken breast cooked at one-step temperature treatments for 120 and 180 min can be perceived only by an experienced observer (ΔE > 2). It can be inferred that the first-step temperature of 50 °C and the time duration in which it was used in the two-step sous vide treatments had a major impact on the color attributes between the studied sous vide treatments.

#### 3.2.3. Texture Properties

Shear force is the most common instrumental method to evaluate the tenderness of cooked meat. Shear force and texture profile analysis (TPA) parameters of the studied sous vide treatments on the chicken breast are displayed in Table 5. Two-way ANOVA revealed significant total treatment time (*p* < 0.01) and treatment time ratio effects (*p* < 0.001) on shear force with insignificant interaction effect (*p* > 0.05). Chicken breasts cooked with the two-step temperature had significantly lower shear force values than chicken breasts cooked with the one-step temperature in the case of 180-min treatments (*p* < 0.05). These results showed the effect of the first-step temperature (50 °C) applied in the 180-min two-step temperature treatments on increasing the tenderness of sous vide chicken breast. In agreement with this result, Hwang et al. [46] reported that shear force values were lower when pork loins were cooked at 50 °C compared to those cooked at 55 or 60 °C. This tenderization effect might be attributed to the specific proteolytic enzymes for desmin degradation, which are largely active up to 50 °C [29]. Chicken breasts cooked with the one-step sous vide obtained higher shear values because at 60 °C the main proteolytic enzymes, calpains, are inactivated [61] while cathepsins operate with only 50% of their proteolytic activity [62].

The results of TPA parameters are shown in Table 5. The two-way MANOVA overall result was significant for both factors (total treatment time: Wilk’s lambda = 0.04, *p* < 0.001; time ratio: Wilk’s lambda = 0.03, *p* < 0.001) with significant interaction (*p* < 0.001). The follow-up univariate ANOVA using Bonferroni’s correction revealed significant results for cohesiveness, gumminess, and chewiness in the case of total treatment time (*p* < 0.05) and an insignificant result for hardness and springiness (*p* > 0.38). Univariate ANOVA resulted in a significant time ratio effect for hardness, gumminess, and chewiness (*p* < 0.05) and it was insignificant for cohesiveness and springiness (*p* > 0.23). The interaction was significant for cohesiveness, gumminess, and chewiness (*p* < 0.05).

The highest TPA-hardness mean value (49.78 N) was obtained in chicken breasts cooked with the 120-min one-step sous vide (T1), which was significantly higher compared to chicken breast cooked with the 120-min two-step sous vide (T2 and T3) (*p* < 0.05). However, hardness was not significantly different in the 180-min two-step sous vide treatments (T5 and T6) compared to the 180-min one-step sous vide treatment (T4). On the other hand, no significant difference was observed in hardness between chicken breast cooked with the one-step sous vide at 60 °C for 120 min (T1) and 180 min (T4). Similarly, Park et al. [56] found that the hardness of chicken breast was not affected by cooking time when cooked at the temperature of 60 °C for different time durations (60, 120, and 180 min). No significant differences were observed between one-step and two-step sous vide on cohesiveness and springiness within the same total treatment time (*p* > 0.05) (Table 5). Previous studies have also reported no difference in springiness and cohesiveness of the sous vide chicken breast [56] and beef [11] cooked at different combinations of temperature and time durations. In addition to hardness, two other important texture parameters especially for elderly consumers are chewiness and gumminess. According to the results presented in Table 5, gumminess was significantly reduced in the two-step sous vide-treated samples in comparison with the one-step sous vide-treated ones within the same total treatment time, except for T5. The trends for gumminess values were just slightly different from chewiness values in sous vide chicken breast treated at the same total treatment time. The chewiness was also reduced in the case of the 120-min two-step sous vide-cooked chicken breast with T2 treatment having a significant decrease. The reduced shear force and hardness (initial bite tenderness) and the reduced gumminess and chewiness obtained in two-step sous vide-treated chicken breast present important texture attributes for an increase in meat consumption by elderly consumers [63].

### 3.3. Lipid Oxidation

To evaluate the rate of oxidation in sous vide-treated chicken breast, the level of the secondary lipid oxidation compounds such as aldehydes (like malonaldehyde) was measured using a TBARS test. This method is used to indirectly detect the level of oxidative rancidity in meat products. The results of TBARS values of sous vide-treated chicken breast with different combinations of temperatures and time durations are shown in Table 6. Two-way ANOVA revealed significant total treatment time and treatment time ratio effects on TBARS (*p* < 0.001) with insignificant interaction effect (*p* = 0.83). Increasing the total treatment time from 120 min to 180 min within the same treatment time ratio caused an increase in TBARS values of the cooked chicken breast. In agreement with our result, Karpinska-Tymoszczyk et al. [17] reported that increasing the cooking time from 90 to 150 min at 61 °C increased the TBARS values of sous vide chicken breast fillets. Chicken breasts treated with the one-step temperature treatments obtained significantly higher TBA values than those treated with the two-step temperature treatments within the same total treatment time (*p* < 0.05) (Table 6). This might be due to the longer application time of the end step temperature in the two-step sous vide treatments, which caused higher protein denaturation, thus higher release of iron ions, which have a strong pro-oxidant effect [64]. The highest mean TBA value (0.520 mg MDA/kg sample) was observed in chicken breasts cooked at 60 °C for 180 min (T4 treatment). According to Akoğlu et al. [65], consumers are not able to detect the oxidative rancidity in sous vide-treated chicken breast with TBARS values below 1 mg/kg. None of the tested two-step sous vide treatments exceeded this threshold level (Table 6).

### 3.4. Protein Solubility

The results for sarcoplasmic, myofibrillar, and total protein solubility of chicken breasts cooked at different combinations of temperatures and time durations are shown in Table 6. The two-way MANOVA overall result was significant for both studied factors (total treatment time: Wilk’s lambda = 0.13, *p* < 0.001; time ratio: Wilk’s lambda = 0.04, *p* < 0.001) with significant interaction (*p* < 0.001). The follow-up univariate ANOVA using Bonferroni’s correction revealed significant results for myofibrillar and total protein solubility in the case of total treatment time (*p* < 0.001) and an insignificant result for sarcoplasmic protein solubility (*p* = 0.64). Univariate ANOVA resulted in a significant time ratio effect for all the three protein solubility variables (*p* < 0.05). The interaction was significant for sarcoplasmic and myofibrillar protein solubility (*p* < 0.05). 

The present study showed a significant decrease in total protein solubility of chicken breast cooked with the one-step sous vide in comparison with the ones cooked with the two-step sous vide within the same total treatment time. Lower total protein solubility indicates the heat denaturation of sarcoplasmic and myofibrillar proteins. Protein denaturation increased with longer cooking time durations of chicken breast at the high temperature of 60 °C, which is in agreement with the results of Cropotova et al. [66]. Sarcoplasmic protein solubility of chicken breasts cooked with the two-step sous vide was increased at a higher rate than myofibrillar protein solubility in comparison to chicken breasts cooked with the one-step sous vide in the 120-min total treatment time but not in the 180-min (Table 6). There is a relation between protein solubility, moisture content, and cooking loss of meat [67,68]. Murphy and Marks reported a strong correlation between protein solubility and cooking loss in heat-treated chicken breast patties [67]. The results of our study are in accordance with this observation (data not shown). Moisture content was also significantly correlated with both myofibrillar and total protein solubility (R = 0.889 and R = 0.785, both with *p* < 0.001).

## 4. Conclusions

The two-step sous vide technique can provide a valuable cooking alternative for elderly consumers as it reduced the main texture parameters (shear force, hardness, chewiness, and gumminess), but at the same time preserved the water content, redness, and oxidative stability in chicken breasts. Furthermore, significantly lower cooking loss and increased protein solubility values were observed in chicken breast cooked with the two-step sous vide compared to the ones cooked with the traditional one-step sous vide. The present study also found that the treatment time ratio between the first- and second-step temperatures (50 and 60 °C) had a significant effect on cooking loss, lightness, total color difference, gumminess, TBARS, sarcoplasmic, and total protein solubility of sous vide-treated chicken breasts within the same total treatment time. Meanwhile, the total treatment time was significant only for moisture content, cooking loss, and TBARS values of cooked chicken breasts within the same treatment time ratios. Regarding microbiological safety, all the two-step sous vide treatments were able to successfully inactivate the vegetative cells of two main pathogens of interest (*C. perfringens* and *L. monocytogenes*) in chicken breast. As expected, none of the studied sous vide heat treatments were enough to inactivate the *C. botulinum* spores; thus, proper refrigeration storage of these products is required. Future investigations need to be done to evaluate the sensory characteristics and the shelf life of chicken breast cooked with the two-step sous vide to determine consumer acceptance, with special emphasis among elderly consumers.

## Figures and Tables

**Table 1:** Sous vide treatments applied to chicken breasts.

Group	Time at the Temperature of 50 °C (min)	Time at the Temperature of 60 °C (min)	Treatment Time Ratio 50 °C/60 °C	Total Treatment Time (min)
T1	0	120	0:1	120
T2	40	80	1:2	120
T3	60	60	1:1	120
T4	0	180	0:1	180
T5	60	120	1:2	180
T6	90	90	1:1	180

**Table 2:** Means ± standard deviations of pasteurization values ($P_{60}^{6.74}$, $P_{70}^{10}$, $P_{80}^{13}$) of chicken breast (*pectoralis major*) cooked with different combinations of temperatures and time durations.

Total Treatment Time	120 min			180 min		
Treatment Time Ratio	0:1	1:2	1:1	0:1	1:2	1:1
Treatments	T1	T2	T3	T4	T5	T6
$P_{60}^{6.74}\;(\min)$	130.26 ± 3.92	86.68 ± 5.09	67.57 ± 2.73	197.55 ± 7.73	136.17 ± 4.41	99.07 ± 3.70
$P_{70}^{10}\;(\min)$	12.21 ± 1.06	9.31 ± 0.37	7.15 ± 0.68	17.34 ± 1.02	13.50 ± 0.71	10.06 ± 0.52
$P_{80}^{13}\;(\min)$	4.01 ± 0.33	2.92 ± 0.19	2.18 ± 0.12	6.01 ± 0.31	4.47 ± 0.28	3.06 ± 0.16

T1: 60 °C for 120 min; T2: 50 °C for 40 min and 60 °C for 80 min; T3: 50 °C for 60 min and 60 °C for 60 min; T4: 60 °C for 180 min; T5: 50 °C for 60 min and 60 °C for 120 min; T6: 50 °C for 90 min and 60 °C for 90 min. $P_{60}^{6.74}$ pasteurization value with *T_ref_* of 60 °C and z-value of 6.74 °C; $P_{70}^{10}$ pasteurization value with *T_ref_* of 70 °C and z-value of 10 °C; $P_{80}^{13}$ pasteurization value with *T_ref_* of 80 °C and z-value of 13 °C.

**Table 3:** Means ± standard deviations of moisture content, cooking loss, and color attributes of chicken breast cooked with different combinations of temperatures and time durations.

Total Treatment Time	120 min			180 min		
Treatment Time Ratio	0:1	1:2	1:1	0:1	1:2	1:1
Treatments	T1	T2	T3	T4	T5	T6
Moisture content (%)	71.21 ± 0.45 ^Ba^	72.09 ± 0.21 ^Ba^	71.94 ± 0.57 ^Ba^	68.9 ± 0.45 ^Aa^	69.73 ± 0.55 ^Aab^	70.12 ± 0.18 ^Ab^
Cooking loss (%)	13.43 ± 0.20 ^Ab^	11.82 ± 0.49 ^Aa^	11.61 ± 0.40 ^Aa^	16.35 ± 0.92 ^Bb^	14.74 ± 0.39 ^Ba^	12.92 ± 0.86 ^Aa^
L*	80.00 ± 0.24 ^Ab^	78.79 ± 0.30 ^Aa^	78.36 ± 0.23 ^Aa^	80.83 ± 0.05 ^Bc^	78.96 ± 0.22 ^Ab^	78.38 ± 0.21 ^Aa^
a*	1.79 ± 0.30 ^Aa^	2.10 ± 0.17 ^Aa^	2.22 ± 0.17 ^Aa^	1.36 ± 0.2 ^Aa^	1.94 ± 0.18 ^Ab^	2.49 ± 0.22 ^Ac^
b*	9.55 ± 0.18 ^Aa^	11.55 ± 0.39 ^Ab^	13.10 ± 0.13 ^Ac^	9.11 ± 0.34 ^Aa^	12.59 ± 0.39 ^Ab^	12.87 ± 0.65 ^Ab^
ΔE	29.34 ± 0.26 ^Ab^	28.64 ± 0.28 ^Aa^	28.69 ± 0.18 ^Aa^	30.06 ± 0.03 ^Bb^	29.08 ± 0.26 ^Aa^	28.65 ± 0.23 ^Aa^

T1: 60 °C for 120 min; T2: 50 °C for 40 min and 60 °C for 80 min; T3: 50 °C for 60 min and 60 °C for 60 min; T4: 60 °C for 180 min; T5: 50 °C for 60 min and 60 °C for 120 min; T6: 50 °C for 90 min and 60 °C for 90 min. Different superscript letters in the same row mean significant differences. Lower case letters (a, b, c) are for comparison of time ratio effects under the same total treatment time (Tukey’s post hoc test, *p* < 0.05). Upper case letters (A, B) are for comparison of total treatment time effects within the same ratio of step time intervals (*p* < 0.05).

**Table 4:** Total color difference (ΔE) between chicken breasts cooked with different combinations of temperatures and time durations.

Total Color Difference (ΔE)						
Treatments	T1	T2	T3	T4	T5	T6
T1	-					
T2	2.36 (Cat. III)	-				
T3	3.94 (Cat. IV)	1.61 (Cat. II)	-			
T4	1.03 (Cat. II)	3.27 (Cat. III)	4.77 (Cat. IV)	-		
T5	3.22 (Cat. III)	1.07 (Cat. II)	0.84 (Cat. I)	3.99 (Cat. IV)	-	
T6	3.76 (Cat. IV)	1.44 (Cat. II)	0.36 (Cat. I)	4.63 (Cat. IV)	0.85 (Cat. I)	-

T1: 60 °C for 120 min; T2: 50 °C for 40 min and 60 °C for 80 min; T3: 50 °C for 60 min and 60 °C for 60 min; T4: 60 °C for 180 min; T5: 50 °C for 60 min and 60 °C for 120 min; T6: 50 °C for 90 min and 60 °C for 90 min. Latin numbers in the table indicate the total color difference (ΔE) categories: Category I: 0 < ∆E < 1, observer perceives no difference; category II: 1 < ∆E < 2, only the experienced observer perceives the difference; category III: 2 < ∆E < 3.5, the color difference is perceived also by an inexperienced observer; category IV: 3.5 < ∆E < 5, a clear color difference is perceived.

**Table 5:** Means ± standard deviations of shear force and TPA attributes of chicken breast (*pectoralis major*) heated with different combinations of temperatures and time durations.

Total Treatment Time	120 min			180 min		
Treatment Time Ratio	0:1	1:2	1:1	0:1	1:2	1:1
Treatments	T1	T2	T3	T4	T5	T6
Shear force (N)	26.17 ± 2.37 ^Aa^	22.65 ± 1.24 ^Ba^	22.37 ± 1.17 ^Ba^	24.38 ± 2.05 ^Ab^	19.70 ± 0.58 ^Aa^	19.28 ± 0.65 ^Aa^
Hardness (N)	49.78 ± 1.05 ^Ab^	39.84 ± 3.44 ^Aa^	37.79 ± 4.16 ^Aa^	46.45 ± 3.39 ^Aa^	44.55 ± 4.70 ^Aa^	41.08 ± 3.91 ^Aa^
Cohesiveness (-)	0.23 ± 0.01 ^Aab^	0.19 ± 0.01 ^Aa^	0.25 ± 0.03 ^Ab^	0.26 ± 0.02 ^Aa^	0.27 ± 0.02 ^Ba^	0.24 ± 0.02 ^Aa^
Springiness (mm)	1.47 ± 0.11 ^Aa^	1.47 ± 0.09 ^Aa^	1.51 ± 0.04 ^Aa^	1.43 ± 0.08 ^Aa^	1.52 ± 0.15 ^Aa^	1.50 ± 0.05 ^Aa^
Gumminess (N)	11.30 ± 0.30 ^Ab^	7.38 ± 0.64 ^Aa^	9.34 ± 0.10 ^Aa^	12.11 ± 0.32 ^Ab^	11.89 ± 0.23 ^Bb^	9.70 ± 0.11 ^Aa^
Chewiness (N * mm)	16.58 ± 1.45 ^Ab^	10.86 ± 1.65 ^Aa^	14.05 ± 0.33 ^Aab^	17.38 ± 1.37 ^Aab^	18.07 ± 1.88 ^Bb^	14.52 ± 0.64 ^Aa^

T1: 60 °C for 120 min; T2: 50 °C for 40 min and 60 °C for 80 min; T3: 50 °C for 60 min and 60 °C for 60 min; T4: 60 °C for 180 min; T5: 50 °C for 60 min and 60 °C for 120 min; T6: 50 °C for 90 min and 60 °C for 90 min. Different superscript letters in the same row mean significant differences. Lower case letters (a, b) are for comparison of ratio effects under the same total treatment time (Tukey’s post hoc test, *p* < 0.05 or * Games–Howell’s post hoc test). Upper case letters (A, B) are for comparison of total treatment time effects within the same ratio of step time intervals (*p* < 0.05).

**Table 6:** Means ± standard deviations of TBARS and protein solubility parameters of sous vide-treated chicken breast with different combinations of temperatures and time durations.

Total Treatment Time	120 min			180 min		
Treatment Time Ratio	0:1	1:2	1:1	0:1	1:2	1:1
Treatments	T1	T2	T3	T4	T5	T6
TBARS (mg MDA/kg)	0.36 ± 0.01 ^Ac^	0.29 ± 0.01 ^Ab^	0.23 ± 0.02 ^Aa^	0.52 ± 0.03 ^Bb^	0.44 ± 0.02 ^Ba^	0.39 ± 0.03 ^Ba^
Sarcoplasmic protein solubility (g/100 g meat)	2.81 ± 0.16 ^Aa^	3.21 ± 0.06 ^Ab^	3.90 ± 0.09 ^Ac^	2.97 ± 0.07 ^Aa^	3.19 ± 0.07 ^Aa^	3.51 ± 0.14 ^Ab^
Myofibrillar protein solubility (g/100 g meat)	4.20 ± 0.06 ^Ba^	4.26 ± 0.05 ^Ba^	4.22 ± 0.11 ^Aa^	3.19 ± 0.29 ^Aa^	3.71 ± 0.07 ^Ab^	3.94 ± 0.18 ^Ab^
Total protein solubility (g/100 g meat)	7.02 ± 0.13 ^Aa^	7.47 ± 0.10 ^Bb^	8.12 ± 0.15 ^Bc^	6.15 ± 0.31 ^Aa^	6.90 ± 0.13 ^Ab^	7.45 ± 0.13 ^Ac^

T1: 60 °C for 120 min; T2: 50 °C for 40 min and 60 °C for 80 min; T3: 50 °C for 60 min and 60 °C for 60 min; T4: 60 °C for 180 min; T5: 50 °C for 60 min and 60 °C for 120 min; T6: 50 °C for 90 min and 60 °C for 90 min. Different superscript letters in the same row mean significant differences. Lower case letters (a, b, c) are for comparison of ratio effects under the same total treatment time (Tukey’s post hoc test, *p* < 0.05). Upper case letters (A, B) are for comparison of total treatment time effects within the same ratio of step time intervals (*p* < 0.05).

## Data Availability

Not applicable.

## References

[1] Marangoni F., Corsello G., Cricelli C., Ferrara N., Ghiselli A., Lucchin L., Poli A. (2015). Role of poultry meat in a balanced diet aimed at maintaining health and wellbeing: An Italian consensus document. Food Nutr. Res..

[2] Donma M.M., Donma O. (2017). Beneficial effects of poultry meat consumption on cardiovascular health and the prevention of childhood obesity. Med. One.

[3] Rémond D., Machebeuf M., Yven C., Buffière C., Mioche L., Mosoni L., Mirand P.P. (2007). Postprandial Whole-Body Protein Metabolism after a Meat Meal Is Influenced by Chewing Efficiency in Elderly Subjects. Am. J. Clin. Nutr..

[4] Lee J.S., Weyant R.J., Corby P., Kritchevsky S.B., Harris T.B., Rooks R., Rubin S.M., Newman A.B. (2004). Edentulism and nutritional status in a biracial sample of well-functioning, community-dwelling elderly: The health, aging, and body composition study. Am. J. Clin. Nutr..

[5] Zhang J., Bowker B., Yang Y., Pang B., Zhuang H. (2020). Effects of Deboning Time and Thawing Method Interaction on Sensory Descriptive Profiles of Cooked Chicken Breast and Thigh Meat. LWT.

[6] Hong G.-E., Kim J.-H., Ahn S.-J., Lee C.-H. (2015). Changes in Meat Quality Characteristics of the Sous-Vide Cooked Chicken Breast during Refrigerated Storage. Korean J. Food Sci. Anim. Resour..

[7] Zavadlav S., Blažić M., Van de Velde F., Vignatti C., Fenoglio C., Piagentini A.M., Pirovani M.E., Perotti C.M., Bursać Kovačević D., Putnik P. (2020). Sous-Vide as a Technique for Preparing Healthy and High-Quality Vegetable and Seafood Products. Foods.

[8] Ruiz-Carrascal J., Roldan M., Refolio F., Perez-Palacios T., Antequera T. (2019). Sous-Vide Cooking of Meat: A Maillarized Approach. Int. J. Gastron. Food Sci..

[9] Baldwin D.E. (2012). Sous Vide Cooking: A Review. Int. J. Gastron. Food Sci..

[10] Díaz P., Nieto G., Garrido M.D., Bañón S. (2008). Microbial, Physical–Chemical and Sensory Spoilage during the Refrigerated Storage of Cooked Pork Loin Processed by the Sous Vide Method. Meat Sci..

[11] Rinaldi M., Dall’Asta C., Paciulli M., Cirlini M., Manzi C., Chiavaro E. (2014). A Novel Time/Temperature Approach to Sous Vide Cooking of Beef Muscle. Food Bioprocess. Technol..

[12] Botinestean C., Keenan D.F., Kerry J.P., Hamill R.M. (2016). The Effect of Thermal Treatments Including Sous-Vide, Blast Freezing and Their Combinations on Beef Tenderness of M. Semitendinosus Steaks Targeted at Elderly Consumers. LWT.

[13] Mortensen L.M., Frøst M.B., Skibsted L.H., Risbo J. (2012). Effect of time and temperature on sensory properties in low-temperature long-time sous-vide cooking of beef. J. Culin. Sci. Technol..

[14] Christensen L., Ertbjerg P., Aaslyng M.D., Christensen M. (2011). Effect of Prolonged Heat Treatment from 48 °C to 63 °C on Toughness, Cooking Loss and Color of Pork. Meat Sci..

[15] Bıyıklı M., Akoğlu A., Kurhan Ş., Akoğlu İ.T. (2020). Effect of Different Sous Vide Cooking Temperature-Time Combinations on the Physicochemical, Microbiological, and Sensory Properties of Turkey Cutlet. Int. J. Gastron. Food Sci..

[16] Głuchowski A., Czarniecka-Skubina E., Buła M. (2020). The Use of the Sous-Vide Method in the Preparation of Poultry at Home and in Catering—Protection of Nutrition Value Whether High Energy Consumption. Sustainability.

[17] Karpińska-Tymoszczyk M., Draszanowska A., Danowska-Oziewicz M., Kurp L. (2020). The Effect of Low-Temperature Thermal Processing on the Quality of Chicken Breast Fillets. Food Sci. Technol. Int..

[18] Ji D.-S., Kim J.-H., Yoon D.-K., Kim J.-H., Lee H., Cho W.-Y., Lee C.-H. (2019). Effect of Different Storage-Temperature Combinations on Longissimus Dorsi Quality upon Sous-Vide Processing of Frozen/Thawed Pork. Food Sci. Anim. Resour..

[19] Ashie I.N.A., Sorensen T.L., Nielsen P.M. (2002). Effects of Papain and a Microbial Enzyme on Meat Proteins and Beef Tenderness. J. Food Sci..

[20] Christensen M., Tørngren M.A., Gunvig A., Rozlosnik N., Lametsch R., Karlsson A.H., Ertbjerg P. (2009). Injection of marinade with actinidin increases tenderness of porcine M. biceps femoris and affects myofibrils and connective tissue. J. Sci. Food Agric..

[21] Zhu X., Kaur L., Staincliffe M., Boland M. (2018). Actinidin Pretreatment and Sous Vide Cooking of Beef Brisket: Effects on Meat Microstructure, Texture and in Vitro Protein Digestibility. Meat Sci..

[22] Naqvi Z.B., Campbell M.A., Latif S., Thomson P.C., McGill D.M., Warner R.D., Friend M.A. (2021). Improving tenderness and quality of M. biceps femoris from older cows through concentrate feeding, zingibain protease and sous vide cooking. Meat Sci..

[23] Pawar V.D., Mule B.D., Machewad G.M. (2007). Effect of marination with ginger rhizome extract on properties of raw and cooked chewon. J. Muscle Foods.

[24] Naveena B.M., Mendiratta S.K. (2005). The tenderization of bufalo meat using ginger extract. J. Muscle Foods.

[25] Moon S.S. (2018). Effect of proteolytic enzymes and ginger extract on tenderization of m. pectoralis profundus from Holstein steer. Korean J. Food Sci. Anim. Resour..

[26] Bhaskar N., Sachindra N.M., Modi V.K., Sakhare P.Z., Mahendrakar N.S. (2006). Preparation of proteolytic activity rich ginger powder and evaluation of its tenderizing effect on spent-hen muscles. J. Muscle Foods.

[27] Sullivan G.A., Calkins C.R. (2010). Application of Exogenous Enzymes to Beef Muscle of High and Low-Connective Tissue. Meat Sci..

[28] Christensen L., Ertbjerg P., Løje H., Risbo J., van den Berg F.W.J., Christensen M. (2013). Relationship between Meat Toughness and Properties of Connective Tissue from Cows and Young Bulls Heat Treated at Low Temperatures for Prolonged Times. Meat Sci..

[29] Ertbjerg P., Christiansen L.S., Pedersen A.B., Kristensen L. The effect of temperature and time on activity of calpain and lysosomal enzymes and degradation of desmin in porcine longissimus muscle. Proceedings of the 58th International Congress of Meat Science and Technology.

[30] BC Centre for Disease Control 2016-Guidelines for Restaurant Sous Vide Cooking Safety in British Columbia. http://www.bccdc.ca/search?k=svguidelines_finalforweb.pdf.

[31] Juneja V.K., Marmer B.S. (1998). Thermal Inactivation of Clostridium Perfringens Vegetative Cells in Ground Beef and Turkey as Affected by Sodium Pyrophosphate. Food Microbiol..

[32] ECFF (European Chilled Food Federation) (2006). Recommendations for the Production of Prepackaged Chilled Food.

[33] SVAC (Sous Vide Advisory Committee) (1991). Code of Practice for Sous Vide Catering Systems.

[34] AOAC (2005). AOAC Official Methods of Analysis.

[35] Commission Internationale de L’Eclairage (CIE) (1986). Colorimetry.

[36] Knispel G. (1991). Factors affecting the process of color matching restorative materials to natural teeth. Quintessence Int..

[37] Bourne M.C. (1978). Texture profile analysis. Food Tech..

[38] Dias M.V., Nilda de Fátima F.S., Borges S.V., de Sousa M.M., Nunes C.A., de Oliveira I.R.N., Medeiros E.A.A. (2013). Use of Allyl Isothiocyanate and Carbon Nanotubes in an Antimicrobial Film to Package Shredded, Cooked Chicken Meat. Food Chem..

[39] Ganhão R., Estévez M., Morcuende D. (2011). Suitability of the TBA Method for Assessing Lipid Oxidation in a Meat System with Added Phenolic-Rich Materials. Food Chem..

[40] Oliveira T.L.C., Junior B.R.D.C.L., Ramos A.L., Ramos E.M., Piccoli R.H., Cristianini M. (2015). Phenolic carvacrol as a natural additive to improve the preservative effects of high pressure processing of low-sodium sliced vacuum-packed turkey breast ham. LWT-Food Sci. Technol..

[41] Warner R.D., Kauffman R.G., Greaser M.L. (1997). Muscle Protein Changes Post Mortem in Relation to Pork Quality Traits. Meat Sci..

[42] Bradford M.M. (1976). A Rapid and Sensitive Method for the Quantitation of Microgram Quantities of Protein Utilizing the Principle of Protein-Dye Binding. Anal. Biochem..

[43] Abel T., Boulaaba A., Lis K., Abdulmawjood A., Plötz M., Becker A. (2020). Inactivation of Listeria monocytogenes in game meat applying sous vide cooking conditions. Meat Sci..

[44] Karyotis D., Skandamis P.N., Juneja V.K. (2017). Thermal Inactivation of Listeria Monocytogenes and Salmonella Spp. In Sous-Vide Processed Marinated Chicken Breast. Food Res. Int..

[45] Peck M.W., Goodburn K.E., Betts R.P., Stringer S.C. (2008). Assessment of the potential for growth and neurotoxin formation by non-proteolytic Clostridium botulinum in short shelf-life commercial foods designed to be stored chilled. Trends Food Sci. Technol..

[46] Hwang S.-I., Lee E.-J., Hong G.-P. (2019). Effects of Temperature and Time on the Cookery Properties of Sous-Vide Processed Pork Loin. Food Sci. Anim. Resour..

[47] Offer G., Restall D., Trinick J. (1984). Water-holding in meat. Recent Adv. Chem. Meat.

[48] Sałek P., Przybylski W., Jaworska D., Adamczak L., Zielińska D., Głuchowski A. (2020). The Effects on the Quality of Poultry Meat of Supplementing Feed with Zinc-Methionine Complex. Acta Sci. Pol. Technol. Aliment..

[49] Aaslyng M.D., Bejerholm C., Ertbjerg P., Bertram H.C., Andersen H.J. (2003). Cooking Loss and Juiciness of Pork in Relation to Raw Meat Quality and Cooking Procedure. Food Qual. Prefer..

[50] Zielbauer B.I., Franz J., Viezens B., Vilgis T.A. (2015). Physical Aspects of Meat Cooking: Time Dependent Thermal Protein Denaturation and Water Loss. Food Biophys..

[51] Sánchez del Pulgar J., Gázquez A., Ruiz-Carrascal J. (2012). Physico-Chemical, Textural and Structural Characteristics of Sous-Vide Cooked Pork Cheeks as Affected by Vacuum, Cooking Temperature, and Cooking Time. Meat Sci..

[52] King N.J., Whyte R. (2006). Does It Look Cooked? A Review of Factors That Influence Cooked Meat Color. J. Food Sci..

[53] Hunt M.C., Sorheim O., Slinde E. (1999). Color and Heat Denaturation of Myoglobin Forms in Ground Beef. J. Food Sci..

[54] Da Silva-Buzanello R.A., Schuch A.F., Gasparin A.W., Torquato A.S., Scremin F.R., Canan C., Soares A.L. (2019). Quality Parameters of Chicken Breast Meat Affected by Carcass Scalding Conditions. Asian-Australas. J. Anim. Sci..

[55] Haghighi H., Belmonte A.M., Masino F., Minelli G., Lo Fiego D.P., Pulvirenti A. (2021). Effect of Time and Temperature on Physicochemical and Microbiological Properties of Sous Vide Chicken Breast Fillets. Appl. Sci..

[56] Park C.H., Lee B., Oh E., Kim Y.S., Choi Y.M. (2020). Combined effects of sous-vide cooking conditions on meat and sensory quality characteristics of chicken breast meat. Poult. Sci..

[57] Langsrud S., Sørheim O., Skuland S.E., Almli V.L., Jensen M.R., Grøvlen M.S., Ueland Ø., Møretrø T. (2020). Cooking Chicken at Home: Common or Recommended Approaches to Judge Doneness May Not Assure Sufficient Inactivation of Pathogens. PLoS ONE.

[58] Holownia K., Chinnan M., Reynolds A., Koehler P. (2003). Evaluation of Induced Color Changes in Chicken Breast Meat during Simulation of Pink Color Defect. Poult. Sci..

[59] Mokrzycki W.S., Tatol M. (2011). Colour difference∆ E-A survey. Mach. Graph. Vis..

[60] Tomasević I., Tomović V., Milovanović B., Lorenzo J., Đorđević V., Karabasil N., Đekić I. (2019). Comparison of a computer vision system vs. traditional colorimeter for color evaluation of meat products with various physical properties. Meat Sci..

[61] Dransfield E. (1994). Optimisation of Tenderisation, Ageing and Tenderness. Meat Sci..

[62] Spanier A.M., McMillin K.W., Miller J.A. (1990). Enzyme Activity Levels in Beef: Effect of Postmortem Aging and End-Point Cooking Temperature. J. Food Sci..

[63] Forde C.G., van Kuijk N., Thaler T., de Graaf C., Martin N. (2013). Texture and Savoury Taste Influences on Food Intake in a Realistic Hot Lunch Time Meal. Appetite.

[64] Estévez M., Ventanas S., Heinonen M. (2011). Formation of Strecker Aldehydes between Protein Carbonyls–α-Aminoadipic and γ-Glutamic Semialdehydes–and Leucine and Isoleucine. Food Chem..

[65] Akoğlu I., Bıyıklı M., Akoğlu A., Kurhan Ş. (2018). Determination of the Quality and Shelf Life of Sous Vide Cooked Turkey Cutlet Stored at 4 and 12oC. Rev. Bras. Ciência Avícola.

[66] Cropotova J., Mozuraityte R., Standal I.B., Rustad T. (2019). The Influence of Cooking Parameters and Chilled Storage Time on Quality of Sous-Vide Atlantic Mackerel (Scomber Scombrus). J. Aquat. Food Prod. Technol..

[67] Murphy R.Y., Marks B.P. (2000). Effect of Meat Temperature on Proteins, Texture, and Cook Loss for Ground Chicken Breast Patties. Poult. Sci..

[68] Joo S.T., Kauffman R.G., Kim B.C., Park G.B. (1999). The Relationship of Sarcoplasmic and Myofibrillar Protein Solubility to Colour and Water-Holding Capacity in Porcine Longissimus Muscle. Meat Sci..

